# Meta-Analysis Reveals Compositional and Functional Microbial Changes Associated with Osteoporosis

**DOI:** 10.1128/spectrum.00322-23

**Published:** 2023-04-12

**Authors:** Oluwamayowa S. Akinsuyi, Luiz F. W. Roesch

**Affiliations:** a Institute of Food and Agriculture, Department of Microbiology and Cell Science, University of Florida, Gainesville, Florida, USA; North-West University

**Keywords:** gut, microbiota, osteoporosis, 16S rRNA, batch effect, random forest

## Abstract

Over the past decade, the role of the gut microbiota in many disease states has gained a great deal of attention. Mounting evidence from case-control and observational studies has linked changes in the gut microbiota to the pathophysiology of osteoporosis (OP). Nonetheless, the results of these studies contain discrepancies, leaving the literature without a consensus on osteoporosis-associated microbial signatures. Here, we conducted a comprehensive meta-analysis combining and reexamining five publicly available 16S rRNA partial sequence data sets to identify gut bacteria consistently associated with osteoporosis across different cohorts. After adjusting for the batch effect associated with technical variation and heterogeneity of studies, we observed a significant shift in the microbiota composition in the osteoporosis group. An increase in the relative abundance of opportunistic pathogens *Clostridium sensu stricto*, *Bacteroides*, and *Intestinibacter* was observed in the OP group. Moreover, short-chain-fatty-acid (SCFA) producers, including members of the genera *Collinsella*, *Megasphaera*, *Agathobaculum*, *Mediterraneibacter*, *Clostridium* XIV, and *Dorea*, were depleted in the OP group relative to the healthy control (HC) group. Lactic acid-producing bacteria, including *Limosilactobacillus*, were significantly increased in the OP group. The random forest algorithm further confirmed that these bacteria differentiate the two groups. Furthermore, functional prediction revealed depletion of the SCFA biosynthesis pathway (glycolysis, tricarboxylic acid [TCA] cycle, and Wood-Ljungdahl pathway) and amino acid biosynthesis pathway (methionine, histidine, and arginine) in the OP group relative to the HC group. This study uncovered OP-associated compositional and functional microbial alterations, providing robust insight into OP pathogenesis and aiding the possible development of a therapeutic intervention to manage the disease.

**IMPORTANCE** Osteoporosis is the most common metabolic bone disease associated with aging. Mounting evidence has linked changes in the gut microbiota to the pathophysiology of osteoporosis. However, which microbes are associated with dysbiosis and their impact on bone density and inflammation remain largely unknown due to inconsistent results in the literature. Here, we present a meta-analysis with a standard workflow, robust statistical approaches, and machine learning algorithms to identify notable microbial compositional changes influencing osteoporosis.

## INTRODUCTION

Osteoporosis (OP) is the most common metabolic bone disease associated with aging. Currently, 200 million people have osteoporosis worldwide, with about 44 million in the United States (1). Reports from medical studies reveal that every 50-year-old woman has a 2.8% risk of dying from a hip fracture (2). Moreover, the overall cost of acute and long-term treatment associated with osteoporosis exceeds 10 billion dollars annually in the United States (3). According to a global estimate, the number of OP-related fractures worldwide is expected to rise from the current annual average of 1.9 million to 3.2 million (a 68% increase) by 2040 (4). Therefore, OP reflects a severe global and public health issue that demands immediate attention.

OP is characterized by loss of bone strength and rigidity, reduced bone mass, and breakdown of bone tissue and its microarchitecture, leading to fracture susceptibility (4). There are two forms of osteoporosis: primary OP, due to estrogen deficiency and natural aging, and secondary OP, caused by actors other than aging or postmenopausal status (5). The pathology of OP is associated with the cumulative imbalance between osteoblasts (bone-forming cells) and osteoclasts (bone-breaking cells), favoring bone loss during the bone remodeling process (5–7). Other factors that could contribute to the development of osteoporosis are low body mass index, low calcium and vitamin D intake, high alcohol consumption, and low estrogen level (1, 8). While the exact mechanism linking gut microbiome dysbiosis and osteoporosis is still being uncovered, researchers have reported that dysregulation of the gut microbiome could affect bone quality (9, 10). Some of the hypothesized mechanisms suggested by studies in the literature include increased intestinal permeability, impaired calcium transport, increased T-cell response, and systemic inflammation via cytokine activation (11–13). Therefore, identifying microbial taxa whose changes drive the disease may provide helpful insight into developing alternative therapies and nutritional support to manage the disease. For example, specific antimicrobial drugs could control dysbiosis, resulting in increased pathogenic organisms. In contrast, those resulting in the disappearance of beneficial commensals could be addressed by administering specific probiotics such as Lactobacillus rhamnosus GG, Limosilactobacillus reuteri (also called Lactobacillus reuteri) DSM 17938, Lactobacillus plantarum DSM 9843, and Bifidobacterium lactis Bb-12 (14).

The gut microbiome contains a vast community of microorganisms, including bacteria, fungi, viruses, and protozoa, that are crucial in maintaining the host’s overall health. The predominant gut microbial phyla are *Firmicutes*, *Bacteroides*, *Actinomycetes*, *Proteobacteria*, and *Verrucomicrobia*, with *Firmicutes* and *Bacteroidetes* representing 90% of the gut microbiota (14). Gut bacteria have numerous functions in the gut, including aiding digestion and nutrient absorption, providing resistance to pathogens, maintaining intestinal epithelium, regulating the immune system, and regulating behavior through the gut-brain axis.

Myriads of case-control studies have characterized the gut microbiome of osteoporosis patients, identifying over 30 differentially abundant taxa between the OP and the healthy groups (9, 15–19). In addition, some of these studies have reported a negative correlation between these differentially abundant taxa and bone mineral density (BMD) (10, 18).

Nevertheless, there is enormous variability in the results obtained from various studies, leaving the literature without a consensus on the key taxa associated with OP. For example, conflicting results have been observed for the genera *Clostridium* XIVa, *Lactobacillus*, and *Eggerthella*, as some studies found them to be decreased in the OP group (10, 20). In contrast, others found no differences (17), and few found them to be significantly enriched in the OP group (18, 21). Also, variations in results for the *Firmicutes*/*Bacteroidetes* (F/B) ratio, a widely accepted marker of dysbiosis, have appeared across the literature. Wang et al. (9) reported an increase in the F/B ratio in the OP group relative to the control, while Li et al. (10) reported the ratio to be lower in the OP group than the control group. Thus, it is important to pool these data and reanalyze them to observe if the variation reported in each study is still discernible, even when its data are merged with data from related studies, or would be obscured by technical differences among studies.

The inconsistency in results might arise from the differences in nutrition, geography, lifestyle, study designs, and the methods employed to analyze the 16S rRNA sequence data. To date, no meta-analysis has been conducted comparing the gut microbiomes of people with osteoporosis with those of healthy controls. In this study, we provide the first pooled reanalysis of five studies that characterize the gut microbiome of people using 16S rRNA gene sequencing. A standard workflow, robust statistical approaches, and machine learning algorithms were employed to identify notable compositional changes influencing OP.

## RESULTS

### Study characteristics and selection strategy.

**FIG 1:**
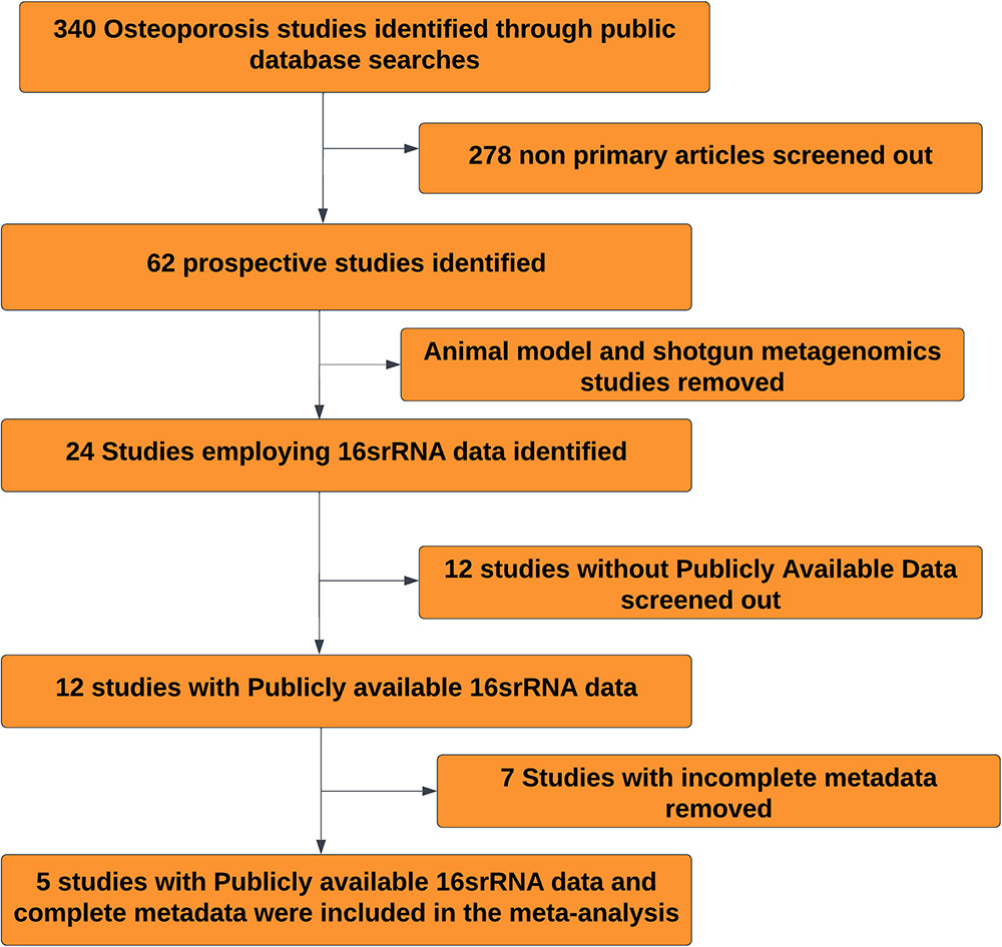
Selection strategy for studies included in the meta-analysis.

Three hundred forty studies, including primary studies, systematic and narrative reviews, and clinical trials, were identified using the search approach (Fig. 1). Among these 340 studies, we identified 62 prospective case-control studies that compared the gut microbiome of the osteoporosis group with that of healthy controls. Of these 62 studies, only 24 characterized the gut microbiome of osteoporosis patients using 16S rRNA gene sequencing. Twelve studies without publicly available data were removed. This left 12 studies that met the inclusion criteria for our analysis. Seven of these studies had incomplete metadata deposited in the SRA (Sequence Read Archive) database. A deliberate effort was made to contact the corresponding authors via email; however, we did not received replies. Therefore, five studies were included in the meta-analysis and processed using the same DADA2 pipeline. Information about each study is found in Table 1. Due to the variation in the primers employed across the five studies, each data set was collapsed at the genus level instead of at the amplicon sequence variant (ASV) level. After collapsing at the genus level, 349 samples were obtained (175 healthy control [HC] and 177 OP samples). However, after rarefaction to 1,500 sequences per sample, a total of 328 samples (155 HC and 173 OP samples) was obtained and used for downstream analysis.

**TABLE 1:** Data sets included in the analysis

Reference	16S rRNA gene region; Illumina sequencing platform	Primers	Age (yrs)	Chemistry	Country	City	Sample size	NCBI accession no.
15	V3-V4; NovaSeq	338F, 806R	≥50	2 × 300	China	Xi’an	85 OP, 28 HC	PRJNA565497
9	V3-V4; MiSeq	338F, 806R	≥64	2 × 300	China	Xi’an	12 OP, 6 HC	PRJNA359375
86	V4; iSeq	541F	NA	1 × 250	South Korea	Asan	16 OP, 60 HC	PRJNA795857
18	V3-V4; MiSeq	341F, 805R	≥60	2 × 250	China	Wuhan	44 OP, 64 HC	PRJNA724901
87	V4-V5; MiSeq	515F, 926R	≥55	2 × 250	China	Wenzhou	24 OP, 18 HC	PRJNA631117

### Alpha diversity and taxonomic composition between osteoporosis and healthy-control groups.

**FIG 2:**
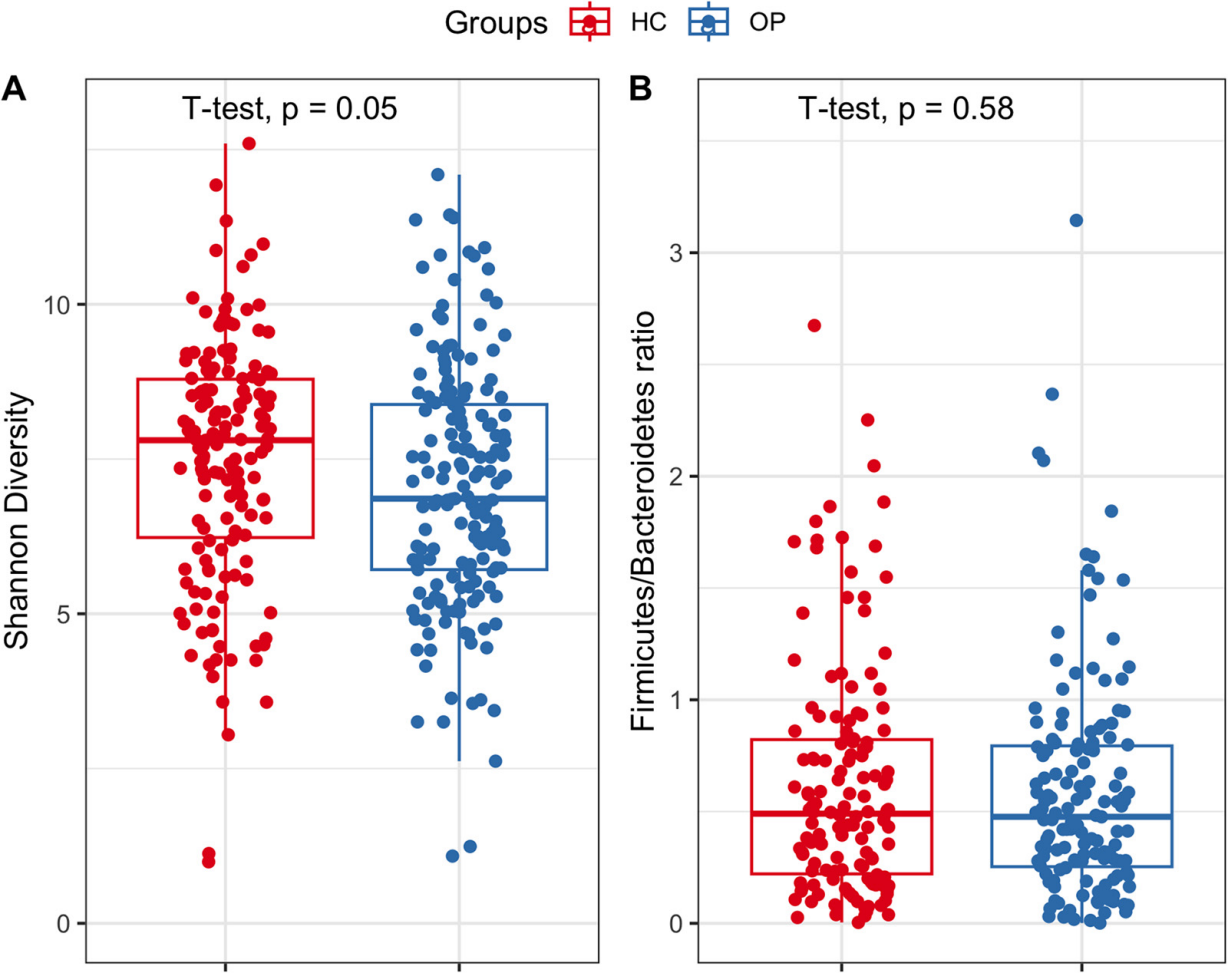
Comparison of alpha (Shannon) diversity (A) and F/B ratio (B) between the OP and HC groups. The boxes span from the first quartile to the third quartile. The horizontal lines inside the boxes depict the medians.

After preprocessing the data, we aimed to address whether gut microbiome diversity is altered in the OP group. We calculated the alpha diversity using the Shannon diversity index. The Shannon diversity index considers the number of species (richness) and evenness. It is a better indicator of diversity when rare and abundant species are considered equally important (22). Our results showed that there is no statistical difference in average Shannon diversity between groups (*P* = 0.05; effect size = 0.17; power = 0.40) (Fig. 2A).

**FIG 3:**
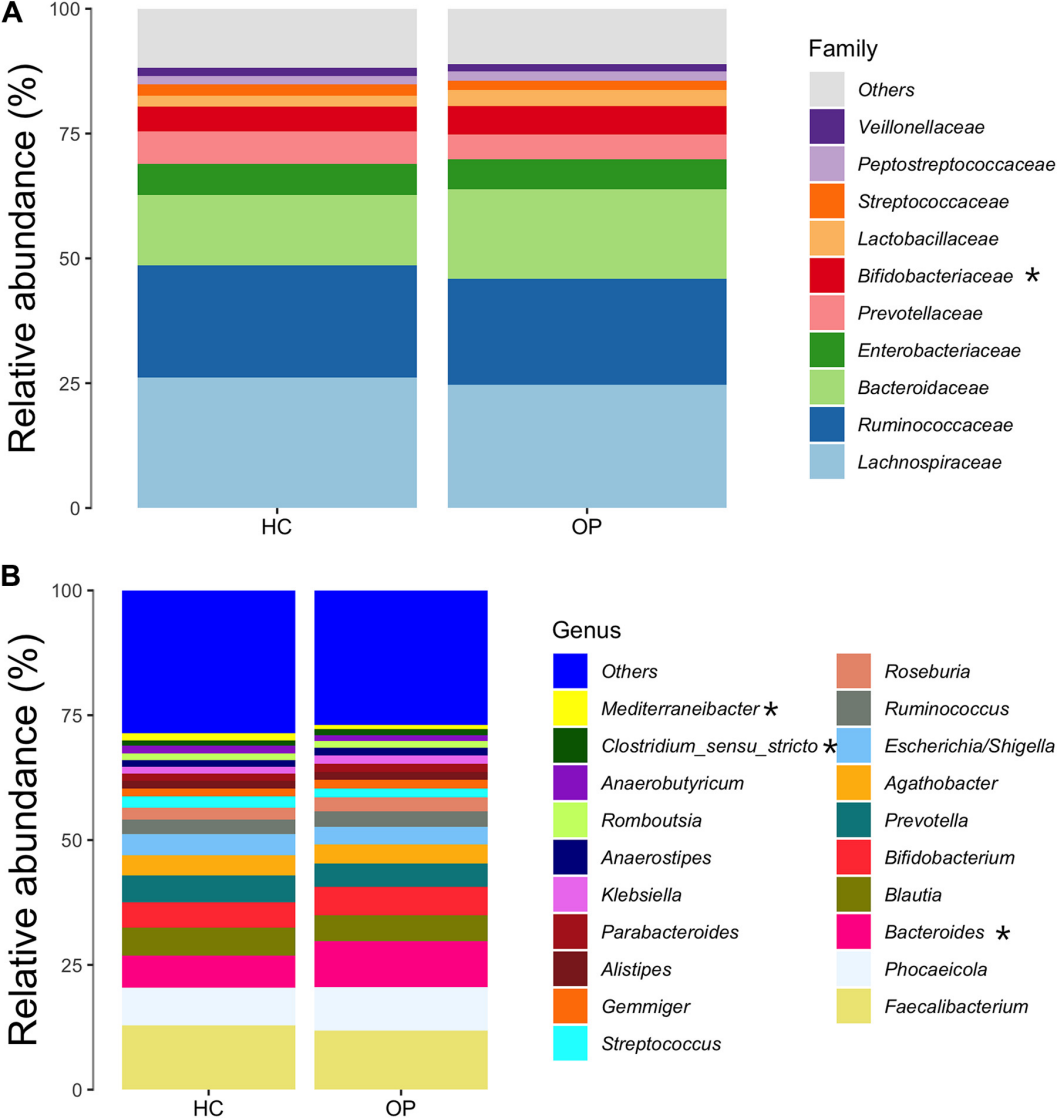
Bacterial taxonomic analysis of gut microbiotas. (A) The 10 most abundant microbial taxa between the OP and the HC groups at the family level. (B) The 20 most abundant microbial taxa between the OP and the HC groups at the genus level. The *x* axis contains information about each group, while the *y* axis represents the relative abundance of each family and genus, respectively. “Others” represents the sum of the relative abundance of all families and genera except those in the figure. Genera and families that statistically differ in relative abundance between groups are marked with an asterisk.

Furthermore, we investigated the changes in composition at various taxonomic rankings. At the phylum level, *Firmicutes*, *Bacteroidetes*, *Proteobacteria*, and *Actinobacteria* were the most dominant phyla in both groups. No significant difference in the F/B ratio between the OP and HC groups was observed (*P* = 0.58) (Fig. 2B). At the family level, *Bacteroidaceae* (*P* = 0.015) was significantly enriched in the OP group (Fig. 3A). At the genus level, *Mediterraneibacter*, a short-chain-fatty-acid (SCFA)-producing genus, was significantly decreased in the OP group (*P* = 0.035). In contrast, *Bacteroides* (*P* = 0.0093) was significantly increased in the OP group. Additionally, members of the genus *Clostridium sensu stricto* were marginally increased in the OP group (*P* = 0.07) (Fig. 3B).

### The structure of the core microbiome community differs between osteoporosis patients and healthy controls.

**FIG 4:**
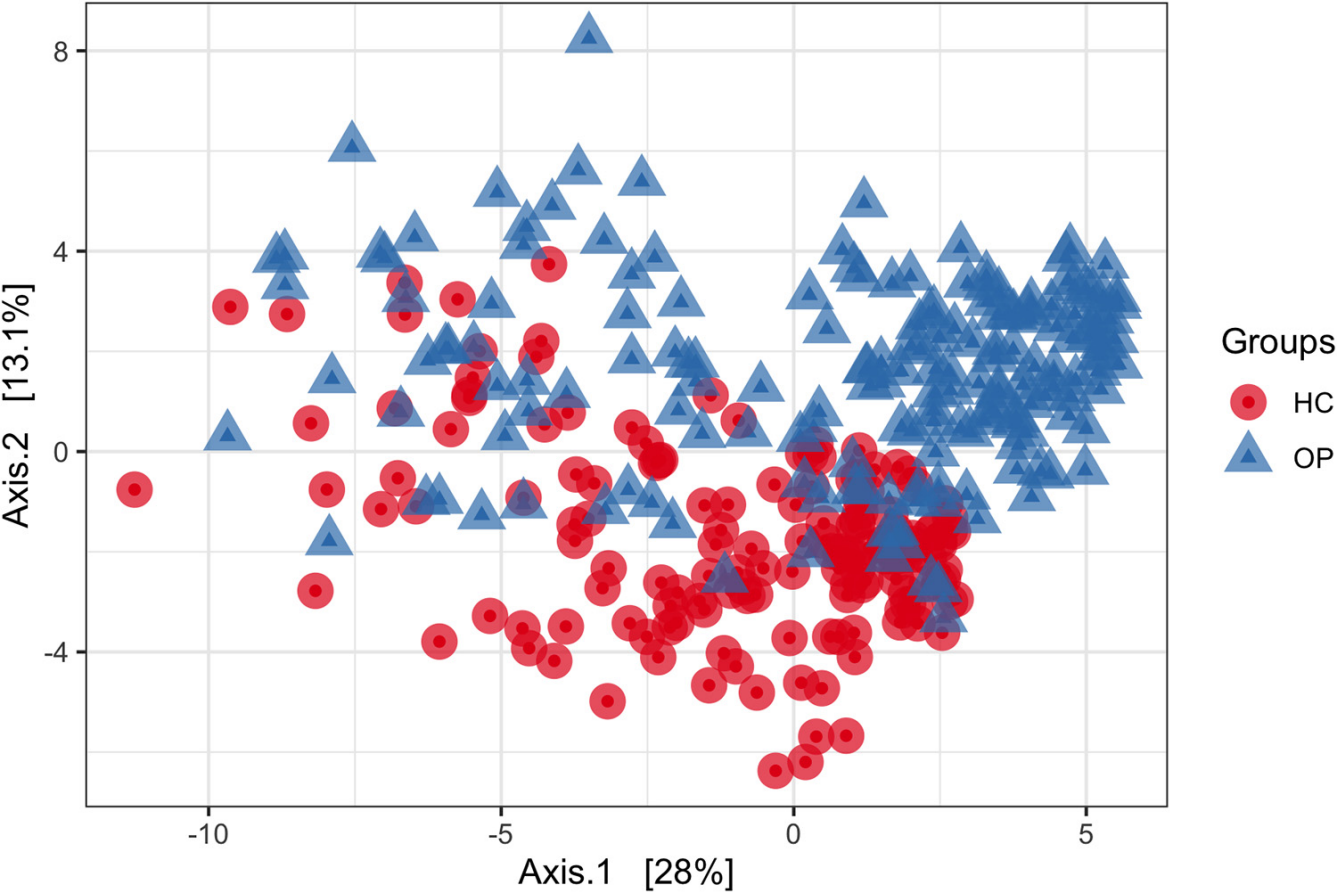
PCoA based on a binomial matrix showing differences in microbial structure between the OP and HC groups at 55% prevalence. Each point represents a microbial community from one sample.

After filtering the data set by prevalence using PIME, we select a 55% cutoff prevalence interval that gives us an out-of-bag (OOB) error of 5% (see Table S2 in the supplemental material). This means that our model is 95% accurate and that the probability of the prediction being by chance is less than 5%. The overall profile of microbial composition between the HC and OP groups at 55% prevalence was visualized using principal-coordinate analysis (PCoA) based on the binomial dissimilarity matrix (Fig. 4). The binomial matrix includes joint absences, thus allowing samples missing the same taxa to appear more similar (23). The PCoA showed a distinctive gut microbial community associated with each group at 55% prevalence. PERMANOVA confirmed differences between the two groups (*R*^2^ = 0.10, *P* = 0.001). The *R*^2^ results suggest that the groups (OP versus HC) explained approximately 10% of the variation in the distance at 55% prevalence. The taxa important to differentiate between the OP and HC was accessed based on mean decrease accuracy as predicted by random forest. Members of the genera *Clostridium sensu stricto*, *Ruminococcus* 2, *Agathobaculum*, *Faecalibacillus*, *Mediterraneibacter*, *Bacteroides*, *Romboutsia*, and *Roseburia*, among others, were important to differentiate between the OP and HC group (Table 2).

**TABLE 2:** Importance of the bacterial genus to differentiate between microbiota samples from subjects with osteoporosis and controls at 55% prevalence

Mean decrease accuracy^*a*^			Gini index	Family	Genus
HC	OP	Over all classes			
0.1507	0.0410	0.0920	28.6566	*Clostridiaceae* 1	*Clostridium sensu stricto*
0.1266	0.0404	0.0809	26.8191	*Lachnospiraceae*	*Ruminococcus* 2
0.0573	0.0739	0.0658	23.7230	*Ruminococcaceae*	*Agathobaculum*
0.1124	0.0189	0.0625	19.6328	*Erysipelotrichaceae*	*Faecalibacillus*
0.1004	0.0147	0.0547	17.8224	*Peptostreptococcaceae*	*Intestinibacter*
0.0045	3.1e^−4^	0.0023	3.0996	*Bacteroidaceae*	*Bacteroides*
−0.0023	0.0006	0.0019	1.8990	*Lachnospiraceae*	*Anaerobutyricum*
−0.0019	0.0041	0.0013	2.0622	*Peptostreptococcaceae*	*Romboutsia*

a A high mean decrease in accuracy shows the taxon’s importance in driving differences between the osteoporosis (OP) and control (HC) groups. The Gini index measures how accurately each variable (OP and HC) affect the homogeneity of nodes and leaves in the random forest model. A higher value signifies higher importance of the taxon to differentiate between the OP and HC groups.

### Differentially abundant taxa between osteoporosis and healthy-control groups.

**FIG 5:**
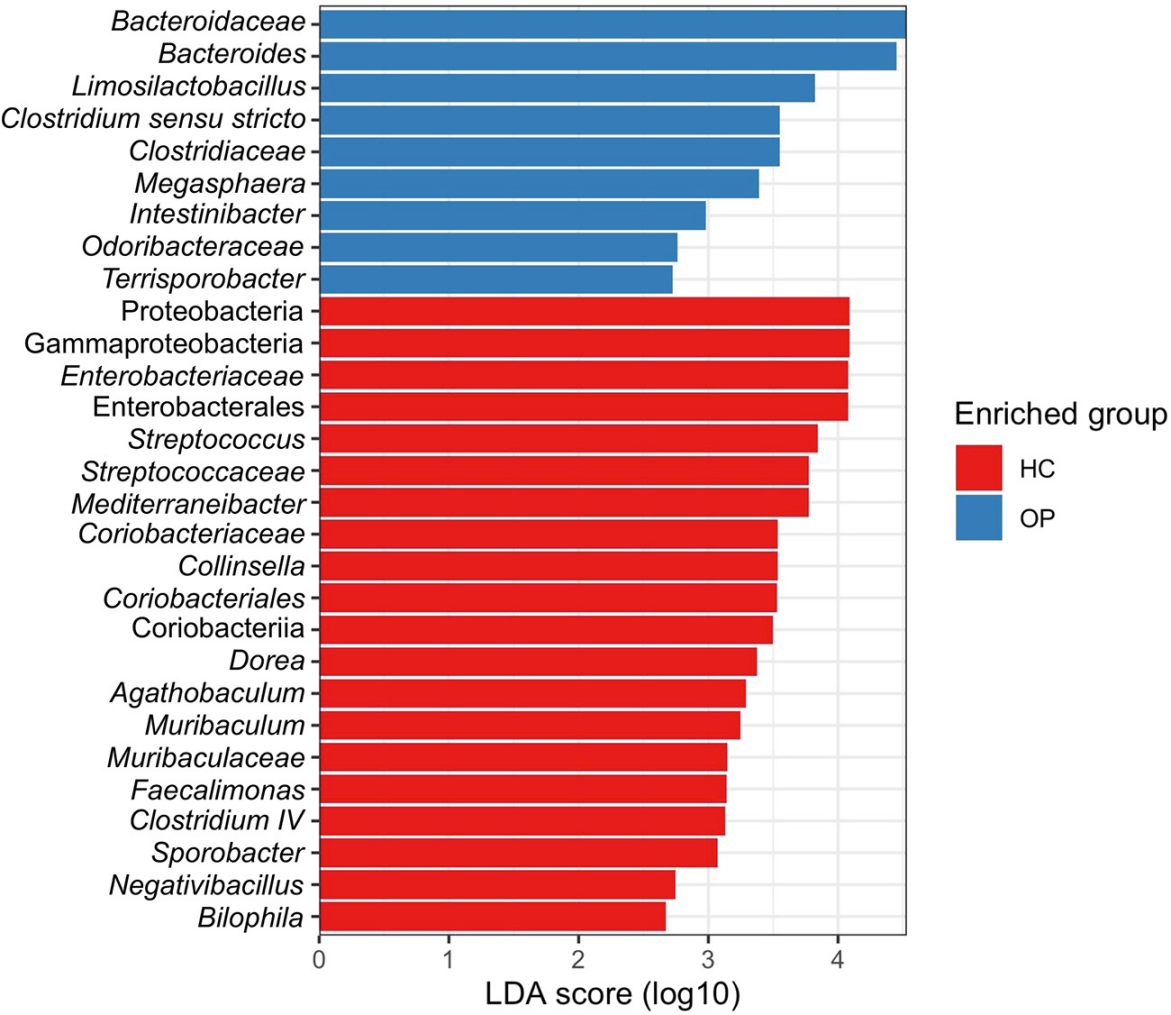
Bar plot showing differentially abundant taxa found to be statistically different between the HC and OP groups, as revealed by LEfSe at a *P* value cutoff of <0.05.

We carried out differential abundance testing to identify signature microbial taxa whose changes could be associated with osteoporosis using the linear discriminant analysis (LDA) combined effect size measurements (LEfSe) (Fig. 5). Members of the families *Bacteroidaceae* and *Clostridiaceae* were significantly increased in the OP group. Members of the genera *Bacteroides*, *Clostridium sensu stricto*, and *Intestinibacter* were more abundant in the OP group. Other genera, such as *Collinsella* and *Megasphaera*, known to produce several SCFAs, including butyrate, acetate, and propionate, were significantly reduced in the OP group. Strict butyrate producers, including members of the genera *Agathobaculum*, *Mediterraneibacter*, *Dorea*, and *Clostridium* XIV, were also decreased in the OP group relative to the HC group. Furthermore, the lactic acid-producing bacterium *Limosilactobacillus* was significantly increased in the OP group.

### Differentially abundant functional pathways between osteoporosis and healthy-control groups.

**FIG 6:**
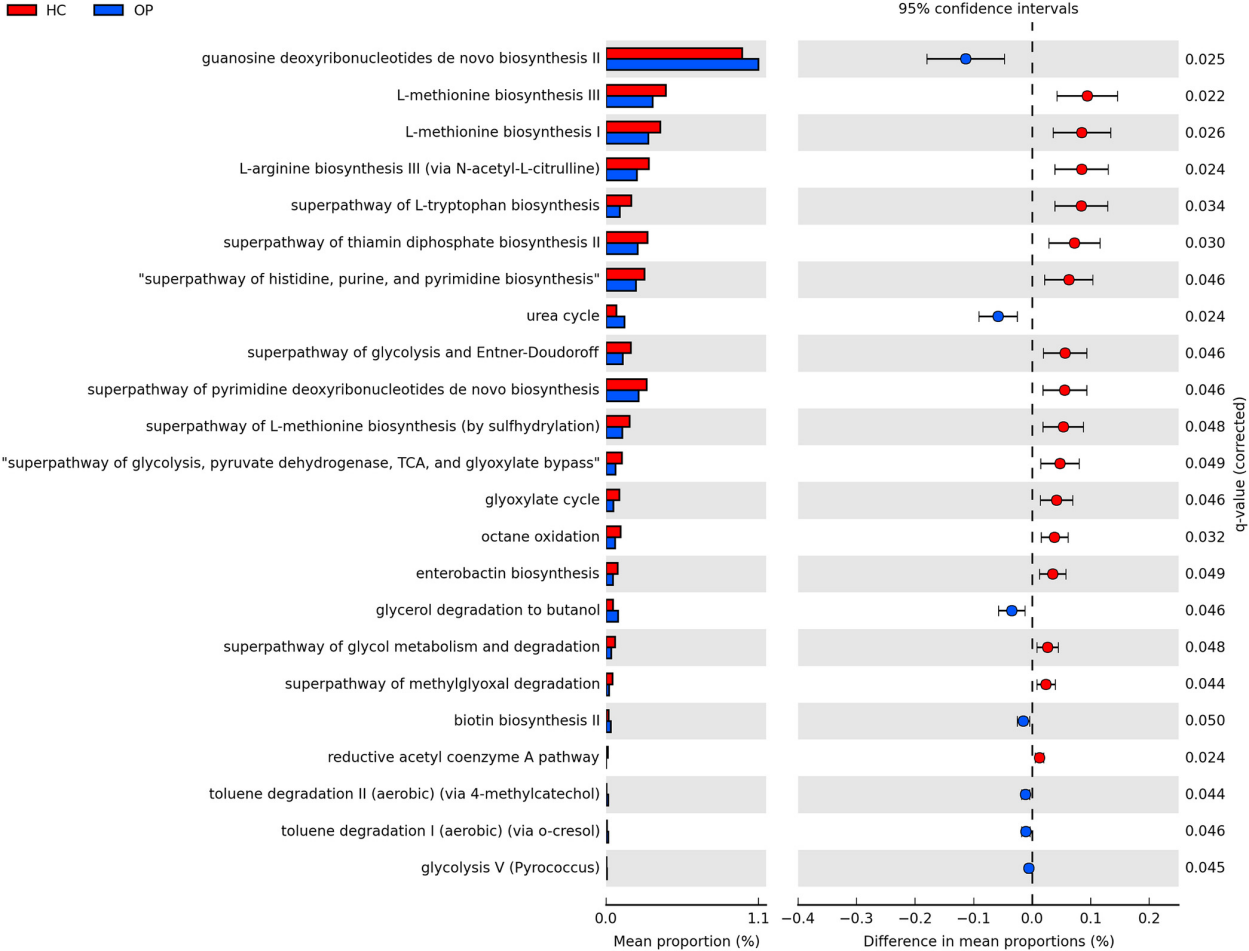
Predicted differential KEGG pathways in the OP and HC groups. The extended error bar plot shows significantly differential KEGG pathways predicted using PICRUSt2 analysis and visualized using the STAMP software. The bar plot on the left depicts each KEGG pathway’s mean proportion. The circles on the left show the difference in mean population between the two groups (effect size). Only *P* values of <0.05 based on Welch’s test are shown.

PICRUSt2 analysis was employed to predict the gut microbial functional pathway to examine if the changes associated with different microbial taxa lead to possible functional changes (Fig. 6). There were 16 pathways significantly enriched in the OP group. These included pathways involved in butyrate production (reductive acetyl-coenzyme A pathways), sugar fermentation to produce SCFAs (glycolysis, tricarboxylic acid [TCA] cycle, and glyoxylate cycle), and amino acid production (l-methionine biosynthesis I and II pathways, and l-arginine biosynthesis III pathways). These amino acids have been reported to ameliorate intestinal inflammation by suppressing nuclear factor κB (NF-κB) and mitogen-activated protein kinase (MAPK) pathways associated with osteoporosis (24, 25). Seven pathways were significantly enriched in the OP groups (*P* < 0.05). These include the urea cycle, biotin synthesis, and toluene degradation pathways, among others. Differentially abundant KEGG enzymes between the OP and HC groups are shown in Fig. S5.

## DISCUSSION

Osteoporosis is a metabolic disease affecting many elderly persons and is a major public health concern. Several studies in the past 5 years have linked shifts in the gut microbiome composition to an increased risk of developing osteoporosis. These studies have presented discordant findings, highlighting the need for a cross-study comparison to identify compositional changes driving the disease.

Microbiome dysbiosis leading to impaired intestinal immune responses and subsequent production of osteoclastogenic cytokines has been proposed as the mechanism by which gut microbes are associated with osteoporosis. To the best of our knowledge, this is the first meta-analysis to reanalyze all publicly available 16S rRNA data comparing the gut microbiome of subjects with osteoporosis and health controls while also placing a strong emphasis on the importance of making raw data and associated patient metadata available for more in-depth analysis in the future. Most studies that have characterized the gut microbiome of osteoporosis patients were conducted in Asia (mostly Japan, China, and Korea). However, a total of three other studies have been conducted in other regions. One study in New Zealand, one in Ireland, and one in the United States (16, 21, 26–29). Our analysis does not include all studies due to the unavailability of data and metadata, limiting the statistical power of the current study. Future reanalysis should build on this study by incorporating cohorts from other populations with adequate statistical power to identify universal osteoporosis-associated signatures. At the same time, the heterogeneity of studies and batch effects have been the major drawbacks hindering the identification of a true association between microbes and disease in most meta-analyses. Our study addresses this issue by employing MMUPHin, a batch effect correction algorithm that reduces technical variation between microbiome studies and identifies microbial changes that could potentially drive osteoporosis. Also, we merged and analyzed our data at the genus level, which inevitably reduced our resolution and interpretation of relationships. Hence, our result should be interpreted carefully, because different species or strains within a genus may have different associations with disease. Furthermore, this study is an association study and thus does not provide causal evidence for the role of the gut microbial community in osteoporosis.

Despite the limitation of our study, our findings provide a more subtle understanding of osteoporosis-associated dysbiosis. Alpha diversity is a holistic estimator of the gut microbiota that measures sample diversity based on the relative abundance of taxa. It is commonly used to identify richness (number of taxonomic groups) and evenness (distribution of the abundances of the microbial groups). Shannon diversity is the most common alpha diversity, a metric that considers the richness and evenness of the microbial taxa present in a community (30, 31). It has been shown to reduce the compositional bias relative to other diversity metrics like observed operational taxonomic units (OTUs), particularly when comparing data from different sources (32). A high Shannon index (H) value represents a diverse and equally distributed community, while a lower value indicates a less diverse community and dominance by a single taxon. Previous studies have shown that a loss or reduction in diversity has been associated with myriads of metabolic diseases. Thus, microbial diversity has emerged as a widely employed indicator of gut microbiome health. From the literature, contrasting results in diversity have been reported in the case of osteoporosis. For instance, reports from Wang et al. (9, 15) show an increase in the Shannon diversity in the OP group compared to HC. In contrast, He et al. (19) reported a significant reduction of diversity in the OP group relative to the HC groups. Conversely, we observed no significant difference in the average Shannon diversity in the OP group compared to the HC group (*P* = 0.049, effect size = 0.17, power = 0.40), consistent with previous findings (17, 21, 28, 33). Most studies comparing the Shannon diversity between the OP and HC groups are underpowered and report only *P* values. Reporting only *P* values does not provide qualitative information on how clinically relevant the difference is, leaving the main question of whether the reduction in diversity drives osteoporosis unanswered. The *P* value is more dependent on effect size and sample size, and a significant *P* value is likely to be found even when the difference between groups is negligible (34). However, the effect size is independent of the sample size. Thus, the *P* value could be confounded based on their dependence on effect size and sample size, and sometimes, a statistically significant result means that a large sample size was used (34, 35). As a result, in the present study, we employed effect size. Although significant, we argued that the *P* value is clinically and biologically meaningless because of the negligible difference in average Shannon diversity between groups (effect size). Hence, future reanalysis of studies with large statistical power to detect a large difference in the Shannon diversity is essential to critique our findings and ascertain if a change in Shannon diversity could be a putative biomarker for osteoporosis.

The *Firmicutes*/*Bacteroides* ratio, a microbial measurement at the phylum level, has been extensively employed as an indicator of gut microbiome dysbiosis in many metabolic diseases, including osteoporosis. Different studies have presented contradictory results for the F/B ratio. For instance, Wang et al. (9) reported an increase in the F/B ratio in the OP group relative to the control group, while Li et al. (10) reported the F/B ratio to be lower in the OP group than the control group. In contrast, we found no significant difference in the F/B ratio in our meta-analysis. Moreover, phylum-level analysis does not provide robust information, because it covers a wide range of pathogenic, commensal, and nonpathogenic organisms with different functions, making it nearly impossible to identify signature organisms associated with the OP. Thus, investigating compositional changes at lower taxonomic levels, such as at family, genus, and species levels, than the F/B ratio could provide sophisticated information on microbial taxa driving the disease.

Compositional microbiota shifts could be affected by several factors, including diet, geography, and genetics, which confirm the assumption that every individual has a unique gut microbiome (36), while still sharing some core microbes. Studies on twins have also shown that microbiota differs even in identical twins (37). To identify core gut microbiome whose changes might be associated with OP, we filtered out taxa with low abundances, leaving only taxa with high relative abundances in most subjects (at least 55% prevalence in the present study). Random forest results showed 27 genera to be important in differentiating between the OP and HC groups. Differential abundance using LEfSe at a cutoff of a *P* value of <0.05 identified seven of these taxa to be significantly different between groups, confirming the result of the random forest prediction. These taxa identified from our studies align with several reports in the literature. For example, in our study, *Clostridium sensu stricto*, an opportunistic pathogen, was significantly increased in the osteoporosis group. Several studies have reported *Clostridium sensu stricto* to cause intestinal inflammation and decrease SCFA production (38–40). The Gram-negative genus *Bacteroides* was significantly increased in the OP group, in agreement with studies by Wei et al. (18) and Rettedal et al. (21). Furthermore, recent studies have found *Bacteroides* negatively correlated with BMD (16, 18). Ma et al. reported *Bacteroides* positively correlated with osteoclastogenesis in rats (41). Findings from *in vivo* and *in vitro* studies also showed that lipopolysaccharide of the Gram-negative membrane promotes bone resorption by impairing osteoclast activities, disrupting the integrity of the intestinal wall and leading to intestinal permeability, which can induce the production of inflammatory cytokines like tumor necrosis factor alpha (TNF-α) and interleukin 1 (IL-1) (42, 43). We conclude that a significant increase in the Gram-negative *Bacteroides* in the OP group could cause bone loss primarily through immune-mediated mechanisms.

*Intestinibacter*, which contains only one species, Intestinibacter bartlettii (after its reclassification from the genus *Clostridium* in 2014 [44]), was increased in the OP group. Consistent with our findings, Wei et al. (18) found *Intestinibacter* to be increased in the osteoporosis group and negatively correlated with BMD. *I. bartlettii* has also been reported to be increased in patients with chronic obstructive pulmonary disease (COPD) (45). COPD is usually associated with secondary conditions like osteoporosis, diabetes, anorexia, fatigue, and inflammatory bowel disease. *Intestinibacter* has been implicated in all these diseases (18, 43, 46–48). Thus, future studies must focus on understanding how this genus might drive the disease.

In addition, reductions in the genera *Agathobaculum* (Agathobaculum butyriciproducens and Agathobaculum desmolans), *Clostridium* XIV, *Collinsella*, *Mediterraneibacter*, and *Dorea*, which containing SCFA-producing species, were found to be significantly reduced in the osteoporosis group. This group of bacteria breaks down carbohydrates to produce SCFAs like butyrate, acetate, and propionate. Hence, a decrease in the relative abundance of these fermentative bacteria results in a corresponding reduction in SCFAs, the primary energy source for gut endothelial cells. SCFAs, particularly butyrate, induce G-protein-coupled receptors, such as GPR41 and GPR43, on the walls of the intestine to aid immune responses (49). They have been reported to interact with GPR43 to suppress the expression of lipopolysaccharide (LPS)-induced cytokines like TNF-α and gamma interferon (IFN-γ) (50), increase the expression of IL-4 and IL-10, and induce Treg cell activation in the colon (51), reduce the production of inflammatory cytokines like NF-κB, and alleviate intestinal inflammation (52, 53). A study reported that butyrate increases the expression of intracellular calcium transporters (54). The expression of these transporters drives an increase in intracellular calcium absorption, which can limit the production of parathyroid hormone and drastically reduce bone resorption (55). All this evidence point to a possible mechanism for how a reduced abundance of SCFA-producing bacteria and their metabolites could be associated with the development of osteoporosis.

Consistent with our findings, reduced butyrate production has been observed in various inflammatory and metabolic diseases like rheumatoid arthritis, type 1 diabetes, and inflammatory bowel disease (56–58). Notably, members of the genus *Collinsella* have been reported to be involved in estrogen metabolism, as they produce β-glucuronidase, an enzyme that converts estrogen from its inactive form to its active form (59). This makes estrogen available for estrogen-dependent physiological processes. The reduction of this genus in the OP group could be a putative factor driving the disease. Moreover, the lactic acid-producing bacterium (LAB) *Limosilactobacillus* was significantly increased in the OP group. Several studies have reported the attenuative properties of LAB on osteoporosis (60–63). Limosilactobacillus reuteri is employed as a probiotic to promote bone health and reduce bone absorption. L. reuteri converts l-histidine from the diet to histamine, which blocks the MEK1/2-ERK1/2 pathway using H2 receptors, ultimately reducing TNF-α production by monocytes (64). The result from our PICRUSt2 analysis corroborates our previous findings that SCFA-producing bacteria are reduced in the OP groups. For example, the acetyl coenzyme A (acetyl-CoA) pathway was reduced in the OP groups. This pathway, also called the Wood-Ljungdahl pathway, is utilized by most of the butyrate-producing bacteria that drive the production of butyrate (65). Furthermore, most of the enzymes and pathways involved in carbohydrate metabolism and transport were decreased in the OP group. This implies a reduction of SCFA synthesis in the OP group, confirming our previous findings. In addition, the biotin synthesis pathway was also found to be increased in the OP group. No extensive correlation between biotin and bone has been reported in the literature. However, studies have shown that it controls the expression of NF-κB through biotinylation. NF-κB induces proinflammatory genes that encode cytokines and chemokines in osteoclastogenesis. A study by Alles et al. (66) showed that suppression of the NF-κB pathway reduces bone resorption in ovariectomized mice. Interestingly, the OP group had a higher concentration of *Bacteroides*, a major gut biotin producer.

Our functional predictions also showed an alteration in amino acid metabolism in the OP group. Reports have shown that some amino acids (arginine, leucine, and isoleucine) improve bone structure and increase the expression of insulin growth factor 1 (IGF-1), an important mediator of osteoblast activities (67). In a study by Jennings et al. (68), it was concluded that high consumption of six amino acids, including alanine, arginine, glutamic acids, leucine, lysine, and proline, significantly increased spine BMD of discordant monozygotic twins. In our study, l-methionine biosynthesis I and II and l-arginine biosynthesis pathways were depleted in the OP group. Bacteria employ these pathways to synthesize methionine and arginine, respectively. Interestingly, arginine and methionine are important precursors of polyamine, including spermidine and spermine. These metabolites have been shown to prevent bone loss via disruption of osteoclastic activation in mice (69). Furthermore, methionine and arginine have been reported to be involved in cartilage formation and bone strengthening (70). For instance, Vijayan et al. reported that methionine prevents induced bone loss by disrupting functional osteoclast development via the Toll-like receptor 4 (TLR-4)/MyD88/NF-κB signaling pathway (70). This finding supports our conclusion and is quite intriguing, suggesting a new avenue for exploring therapeutic options for people with osteoporosis.

### Conclusions.

Our results identified consistent microbial compositional and functional osteoporosis-related changes in five previously published cohort studies. We observed opportunistic pathogens, including *Bacteroides*, *Intestinibacter*, and *Clostridium sensu stricto*, to be enriched in the OP group. Furthermore, our findings revealed alterations in carbohydrate metabolism (glycolysis, reductive acetyl coenzyme, and glyoxylate pathways) and a decrease in the relative abundance of SCFA-producing bacteria, including *Agathobaculum*, *Dorea*, *Clostridium* XIV, *Collinsella*, and *Mediterraneibacter*, as a key feature possibly driving osteoporosis. Moreover, the metabolism of amino acids such as tryptophan, methionine, and arginine, which play a crucial role in increasing bone density, was also observed to be disrupted in the OP group.

These findings show that gut microbial dysbiosis in osteoporosis patients is associated with functional changes, which result in significant changes in metabolites that play a key role in bone metabolism. We believe the result of this pooled reanalysis sets the stage for future studies to provide more comprehensive knowledge on how dysbiosis in the gut microbiome contributes to osteoporosis.

## MATERIALS AND METHODS

### Data set selection.

Publicly available databases, including Scopus (https://www.scopus.com), Google Scholar (https://scholar.google.com/), PubMed (https://pubmed.ncbi.nlm.nih.gov/), and Web of Science (https://www.webofscience.com), were searched for studies that contained the keywords “osteoporosis and microbiome” or “16S rRNA and osteoporosis.” This resulted in 340 entries. Each study was manually evaluated to ascertain if it satisfied the inclusion criteria. In addition, public nucleotide databases, including Sequence Read Archive (SRA) (https://www.ncbi.nlm.nih.gov/sra) and European Nucleotide Archive (ENA) (https://www.ebi.ac.uk/ena/browser/home), were searched using the same keywords to identify data sets from unpublished studies.

### Inclusion and exclusion criteria.

We included every study that characterized the gut microbiome of osteoporosis patients by comparing their gut microbiome composition with healthy controls (HC) using 16S rRNA gene sequencing. We incorporated studies with any design, including cohort, case-control, and cross-sectional studies. Studies were excluded if they were: (i) reviews, systematic reviews, or meta-analyses; (ii) *in vitro* or mouse studies; (iii) studies that utilized shotgun metagenomics; (iv) books, book chapters, or dissertations; or (v) not published in English.

### 16S rRNA gene sequencing processing.

Demultiplexed raw DNA sequences from the stools of osteoporosis and healthy subjects from different studies were downloaded from the NCBI SRA. Due to the technical variation in the data sets included in the meta-analysis (DNA extraction kits, primers, sequencing, and platform), each data set was separately denoised and processed into amplicon sequence variants using DADA2 (71). We employed the amplicon sequence variant method provided by DADA2 because it generates fewer false-positive sequence variants than OTU-based methods (71). Also, its resolution of biological differences allows exact sequence inferences (100% identity). The demultiplexed FASTQ file was first visualized using the plotQualityProfile function to access quality. Primers were removed, and the truncated reads were filtered to remove unambiguous nucleotide (N), allowing only 2 expected errors at maximum, removing PhiX reads while still allowing high-quality overlaps between the forward and reverse reads (72). This filtering and denoising step was carried out using the “filterAndTrim” function and performed on each data set following the default parameters on DADA2. DADA2’s machine learning algorithm learned error rates using the learnErrors function. The mergePairs function was used to merge paired-end reads, and chimeras were removed using the consensus method. Taxonomy assignment was performed using the Bayesian RDP classifier trained with the RDP_train_set_18 database (73, 74). ASV and the taxonomic table were generated after DADA2 processing. Each data set’s ASV and taxonomy tables were combined with the appropriate metadata to make a phyloseq object (75). Each phyloseq object was filtered by removing chloroplast/cyanobacterium sequences, keeping only ASVs with at least five sequences. Ultimately, each phyloseq object was collapsed into one object at the genus level for downstream analysis.

### Statistical analysis.

All statistical analyses in the meta-analysis were performed with R version 4.1.3. The batch effect, the variation introduced due to technical differences in sample processing and sequencing, was assessed using the multivariate permutational analysis of variance (PERMANOVA) with the Bray-Curtis distance matrix (Table S1A). MMUPHin (76) was used to reduce the batch effect while preserving the magnitude of biological differences between groups (Table S1B) as performed by previous studies (77–79). All downstream analysis was performed on the batch effect-adjusted abundance count. Alpha diversity, which is the diversity within a particular area or subject, was calculated by the vegan R package (80) using the Shannon diversity index. Because the alpha diversity index is susceptible to uneven sampling depth between samples, the Shannon diversity was calculated after rarefaction to the minimum sampling depth of 1,500 sequences. The Shapiro-Wilk test on base R was used to access the normality of the Shannon diversity index of each group. Since the data were not normally distributed, we assessed whether this resulted from skewness or outliers. We accessed skewness and outliers using the Skewness function on R and a box plot, respectively. Shannon diversity values were transformed using the square transformation to satisfy the normality assumption. Normality was confirmed after data transformation (Fig. S1A and B). A box plot illustrating the Shannon diversity between the two groups was obtained using the ggplot2 R package v1.0.5. The parametric *t* test on the GGPUBR package was used to compute the difference in average Shannon diversity between the two groups. To further determine the magnitude of the Shannon diversity difference between the two groups, Cohen’s effect size was computed using the “effect” function in the Rstatix package in R. Power was calculated using the pwr.t.test function in the pwr package. The F/B ratio, a biomarker of dysbiosis, was also calculated. To calculate this ratio between the two groups, the ASV table was agglomerated at the phylum level using the Tax-glom function in the phyloseq package (75). The F/B abundance ratio in each sample was computed on base R after log transformation to meet the normality assumption. The normality assumption was assessed as described above (Fig. S2A and B). The difference in the F/B ratio between the OP and HC groups was calculated using the parametric *t* test on GGPUBR.

To avoid data sparsity due to the presence of singletons and low-abundance taxa without any biological relevance, we employed the algorithm from the PIME package (81). PIME removed the within-group differences and captured only biologically significant variation with a high sample prevalence. The tool employs a random forest algorithm to determine the best range of core microbiome prevalence, which detects taxa important to differentiate between the comparison groups. The term “important” considers several factors, including relative abundance and prevalence. PIME identified a prevalence interval of 55% as one of the cutoffs with the lowest OOB error rate of 0.05. This prevalence cutoff was employed because, based on the OOB error rate, the probability of the prediction being by chance is less than 5%, which is the standard for most statistical analyses. Thus, this means our predictions are 95% accurate in this work.

Beta diversity, which compares the diversity between two or more subjects, was accessed using PCoA using a binomial distance matrix. Differences in the gut microbiome composition between the OP and the HC group were calculated using PERMANOVA (82) with 999 permutations via the Adonis function on the vegan package on R.

Furthermore, to validate PIME prediction, we assessed the chances of overfitting or underfitting data following the recommendations of Ball et al. (83). This was performed by running the analysis using completely random data as follows: shuffling the original data set’s sample labels (OP or HC) into arbitrary groups using 100 bootstrap iterations and running the pime.error.prediction function at each randomization for each prevalence interval (Fig. S3). The validation step indicated the absence of overfitting or underfitting.

Differential abundance was computed using LEfSe (84), using default recommended settings at an adjusted *P* value of ≤0.05 for significant taxa and an LDA effect size of at least 2 for every significant taxon.

Furthermore, we predicted pathways for each data set through phylogenetic studies by reconstructing unobserved states using PICRUSt2 (30) to examine the functional differences between OP and HC groups. The PICRUSt2 output for each data set was preprocessed in R. The output was ultimately visualized using the STAMP (Statistical Analysis of Taxonomic and Functional Profiles) tool (85) (https://beikolab.cs.dal.ca/software/STAMP) to detect differentially abundant functions between the two groups.

### Data availability.

All data are publicly available at https://github.com/luizroesch/MICROBIAL-CHANGES-ASSOCIATED-WITH-OSTEOPOROSIS.
