# Supplementary material for: Meta-Analysis Reveals Compositional and Functional Microbial Changes Associated with Osteoporosis
Source: Microbiol Spectr. 2023 Apr 12;11(3):e00322-23. doi: 10.1128/spectrum.00322-23 (PMC10269714; doi:10.1128/spectrum.00322-23)
Supplement: Supplemental file 1 — Supplemental material. Download spectrum.00322-23-s0001.pdf, PDF file, 1.4 MB [file spectrum.00322-23-s0001.pdf]

Supplementary Table 1. Batch effect Adjustment.

A) PERMANOVA using Bray Curtis distance matrix, showing the amount of variation in microbial abundance profiles explained by study differences before batch effect adjustment.

	DF	SUM of Sqs	R2	F	Pr(>F)
Study	4	14.305	**0.17369**	16.974	**0.001***
Residual	323	68.054	0.82631		
Total	327	82.360	1.00000		

B) PERMANOVA using bray Curtis showing the amount of variation in microbial abundance profiles explained by study differences after batch effect adjustment.

	DF	SUM of Sqs	R2	F	Pr(>F)
Study	4	3.874	**0.05292**	4.5121	**0.001***
Residual	323	69.325	0.94708		
Total	327	72.199	1.00000		

Supplementary Table 2. Prevalence intervals with the out-of-bag error rate for each prevalence level and genus retained in the dataset and the corresponding number of sequences. The prevalence interval chosen in the present study (55%) is shown in bold.

Prevalence Interval (%)	OOB error rate (%)	Number of Genus	Number of Sequences
5	29.57	142	482791
10	12.5	102	476817
15	19.51	81	470659
20	14.63	70	461847
25	7.01	60	450765
30	6.4	52	442720
35	5.18	46	429694
40	5.49	39	417790
45	14.94	32	407710
50	13.41	30	389336
**55**	**4.88**	**29**	**372372**
60	4.88	27	369135
65	48.78	22	359356
70	4.57	22	340175
75	3.96	17	306147
80	9.76	10	246426
85	2.13	8	218298
90	5.49	6	190350

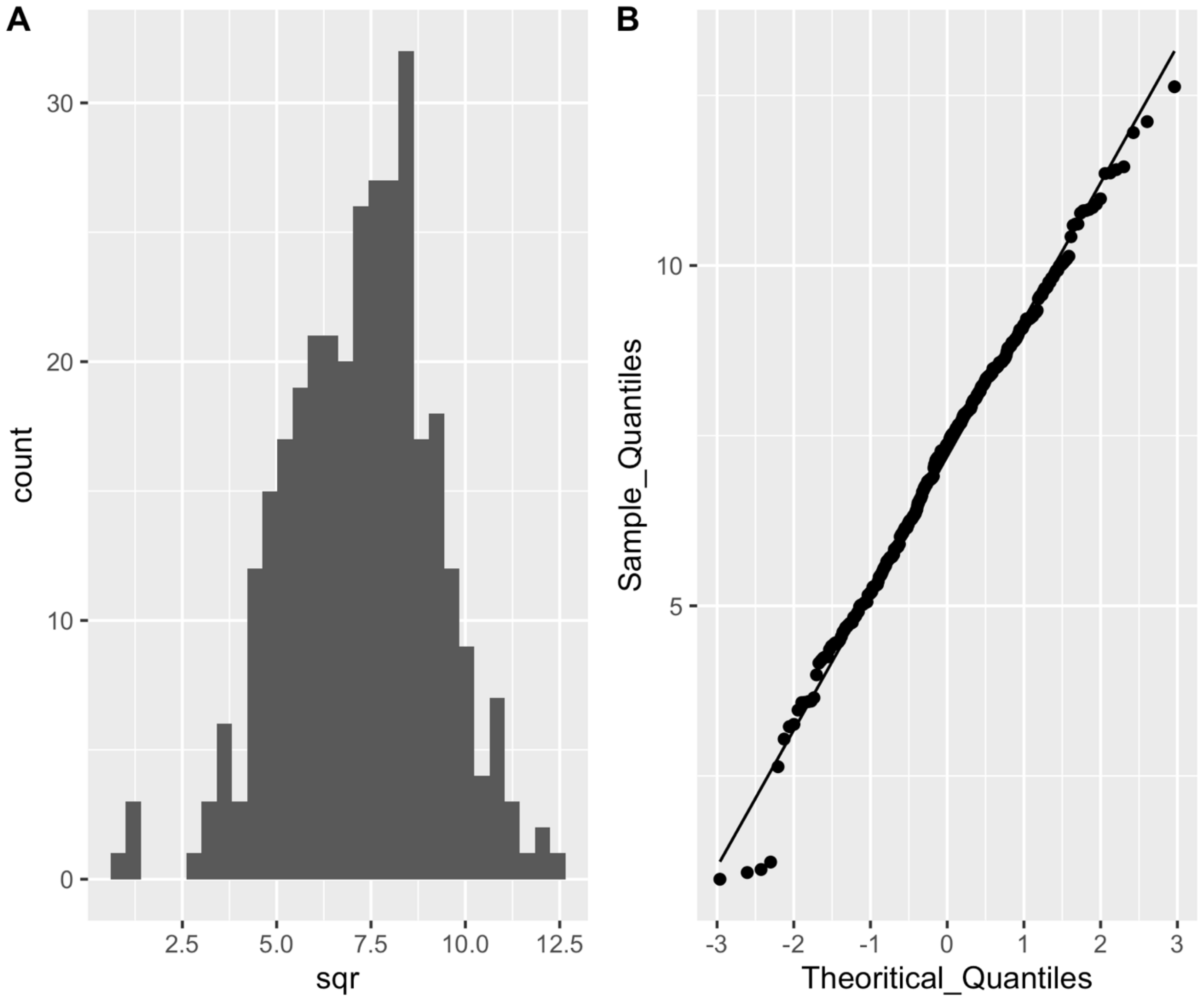

Supplementary Fig. 1: Histogram (A) and qqplot (B) testing the assumption of Normality on Shannon diversity between groups after transforming the data using the square transformation.

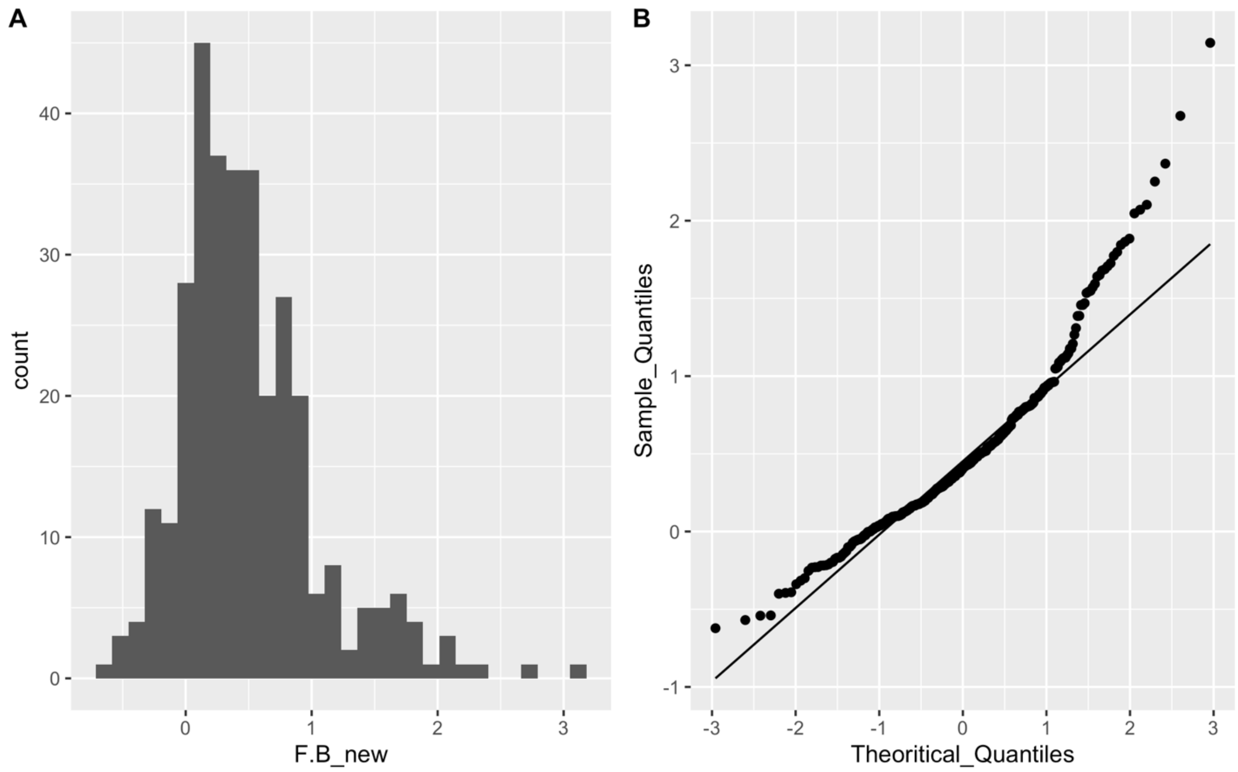

Supplementary Fig. 2: Histogram (A) and qqplot (B) testing the assumption of Normality on Firmicutes/Bacteroidetes ratio(F/B ratio) between groups after logarithm transformation.

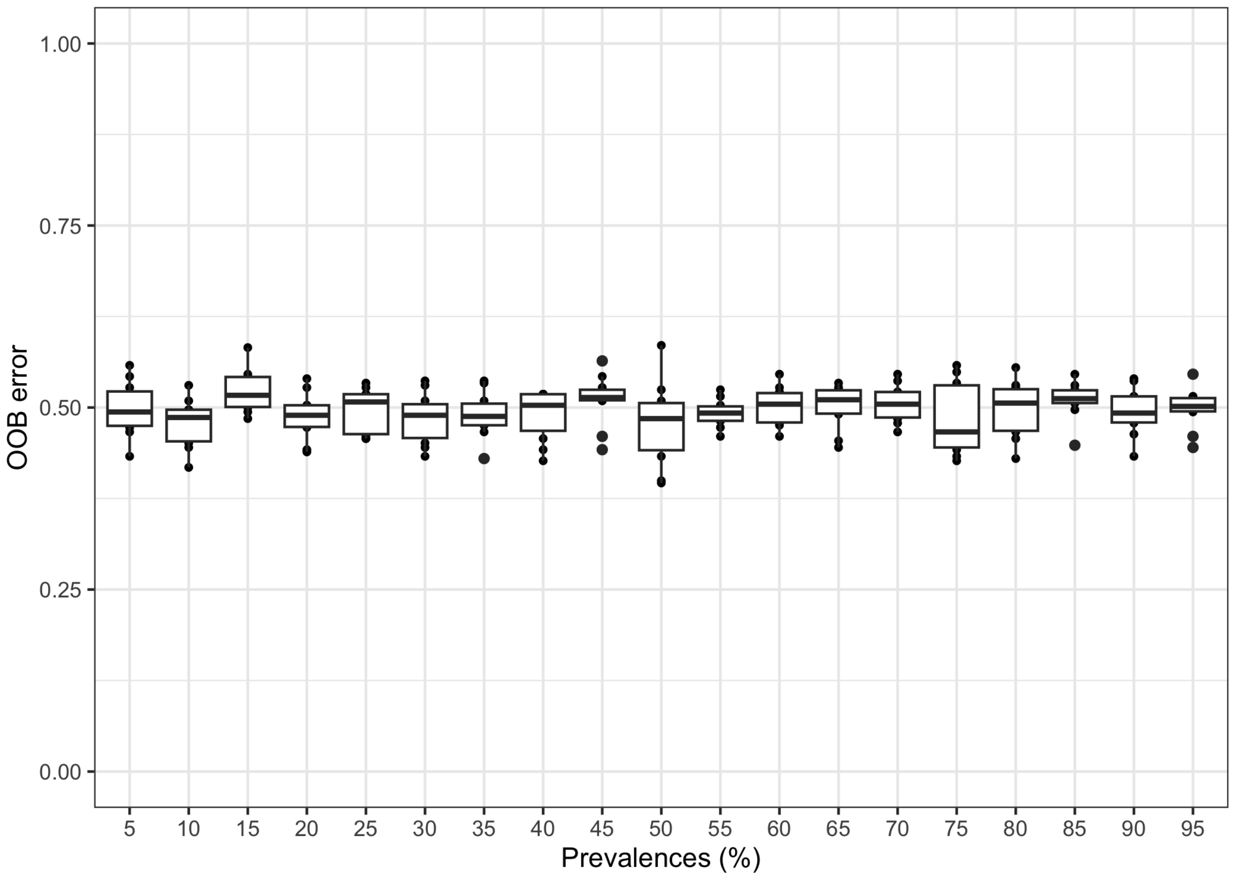

Supplementary Fig. 3: Validation of PIME result by simulating OOB error predictions. Boxplot showing OOB error rate shuffling the original dataset's sample labels (OP or HC) into arbitrary groups using 100 bootstrap iterations for each prevalence interval.

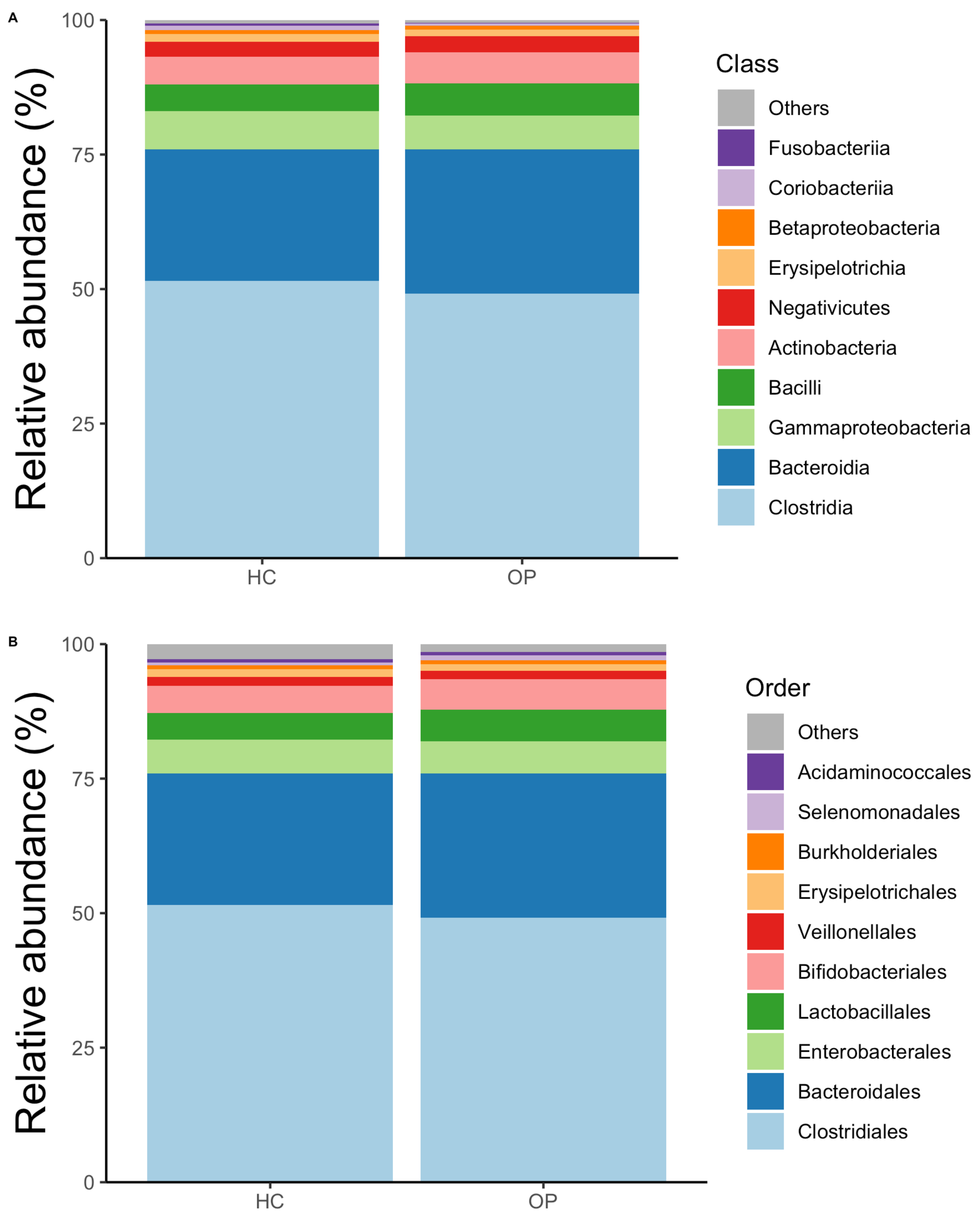

Supplementary Fig. 4: Bacterial taxonomic analysis of gut microbiota. (A) Bar plot showing the most abundant top 10 microbial taxa between the OP and the HC groups at the class level. (B) Bar plot showing the most abundant top 10 microbial taxa between the OP and the HC groups at the

order level. The abscissa contains information about each group, while the coordinate represents the relative abundance of each class and order, respectively. Others represent the sum of the relative abundance of all classes and orders except those in the figure.

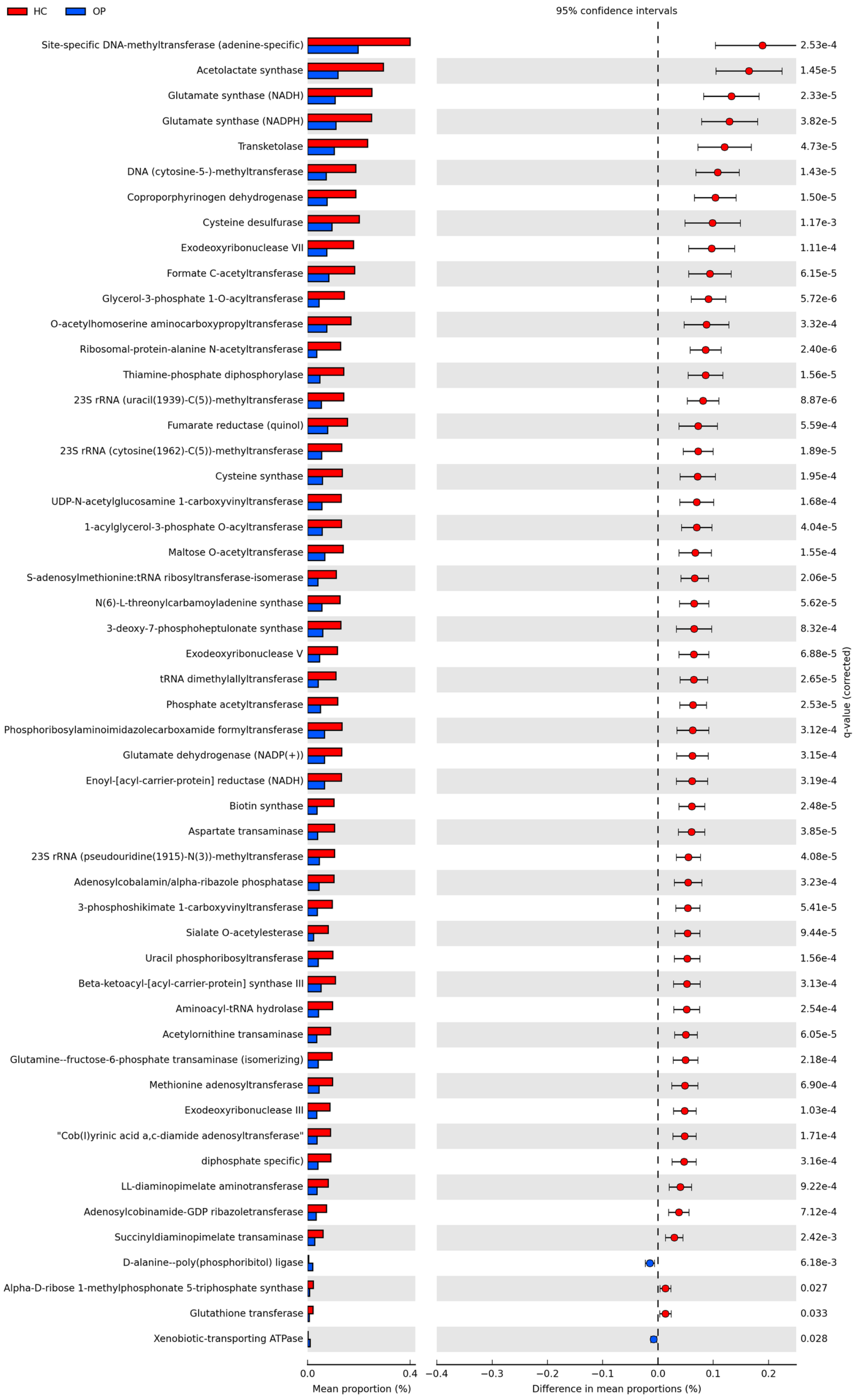

Supplementary Fig. 5: Predicted differential KEGG pathway in OP and HC group. The extended error bar plot depicts significantly differential abundant KEGG enzymes predicted using

PICRUST2 analysis and visualized using the STAMP software. The Bar plot on the left depicts each KEGG pathway's mean proportion. The red and blue bars represent the HC and OP, respectively. The circle on the left shows the difference in mean population between the two groups (effect size). Only p value <0.05 based on Welch's are shown.
